# Supplementary material for: The Association between Loneliness and Health Related Quality of Life (HR-QoL) among Community-Dwelling Older Citizens
Source: Int J Environ Res Public Health. 2020 Jan 17;17(2):600. doi: 10.3390/ijerph17020600 (PMC7013468; doi:10.3390/ijerph17020600)
Supplement: Supplementary file 1 [file ijerph-17-00600-s001.pdf]

Table S1. Participants characteristics, stratified to domains of loneliness.

Items	Emotional loneliness			Social loneliness		
	**Yes (N=627)** Mean±SD N(%)	**No (N=1542)** Mean±SD N(%)	***P-value***	**Yes (N=575)** Mean±SD N(%)	**No (N=1594)** Mean±SD N(%)	***P-value***
Age	80.1±5.8	79.5±5.5	0.021*	80.4±5.3	79.3±5.7	<0.001***
Sex			<0.001***			0.137*
Female	443(33,7)	870(66,3)		363(27,6)	950(72,4)	
Male	184(21,5)	672(78,5)		212(24,8)	644(75,2)	
Country			<0.001***			<0.001***
The United Kingdom	91(17,2)	437(82,8)		71(13,4)	457(86,6)	
Greece	146(44,1)	185(55,9)		74(22,4)	257(77,6)	
Croatia	197(41,0)	284(59,0)		269(55,9)	212(44,1)	
The Netherlands	81(24,1)	255(75,9)		69(20,5)	267(79,5)	
Spain	112(22,7)	381(77,3)		92(18,7)	401(81,3)	
Level of education+			<0.001***			0.017*
Primary or less	206(35,0)	382(65,0)		142(24,1)	446(75,9)	
Secondary or equivalent	371(27,2)	991(72,8)		387(28,4)	975(71,6)	
Tertiary or higher	38(19,7)	155(80,3)		39(20,2)	154(79,8)	
Living situation+			<0.001***			0.060
Living alone	311(23,2)	1029(76,8)		335(25,0)	1005(75,0)	
Living with others	314(38,2)	509(61,8)		236(28,7)	587(71,3)	
Alcohol risk+			0.005**			0.002**
No	459(30,2)	1063(69,8)		424(27,9)	1098(72,1)	
Yes	129(23,8)	414(76,2)		115(21,2)	428(78,8)	
Physical activity+			<0.001***			<0.001***
< once a week	361(23,4)	1184(76,6)		333(21,6)	1212(78,4)	
≥ once a week	259(42,5)	351(57,5)		237(38,9)	373(61,1)	
Smoking*			0.395			0.947
No	575(28,6)	1432(71,4)		532(26,5)	1475(73,5)	
Yes	50(31,8)	107(68,2)		42(26,8)	115(73,2)	
Multi-morbidity+			<0.001***			0.087
No	33(16,8)	164(83,2)		42(21,3)	155(78,7)	
Yes	592(30,1)	1378(69,9)		531(27,0)	1439(73,0)	

SD=standard deviation

+Missing items: Level of education =26; Living situation=6; Lifestyle-Alcohol=104; Lifestyle-Exercise=14; Lifestyle-Smoking=5; Multi-morbidity=2

*p<0.05, **p<0.01, ***p<0.001, P-values are based on Independent T test for participants who are not lonely and participants who are lonely

Table S2. Standardized (full) models of the association between loneliness and Health-Related Quality of Life.

	**PCS-12**			**MCS-12**		
		95%-CI			95%-CI	
	Std-β	lower	upper	Std-β	lower	upper
**Loneliness** (*Yes versus No*)						
Emotional loneliness	-1,39 ***	-1,88	-0,91	-3,73 ***	-4,16	-3,31
Social loneliness	-0,95 ***	-1,44	-0,45	-1,84 ***	-2,27	-1,41
**Age**	-1,49 ***	-2,00	-0,97	0,39	-0,06	0,84
**Sex**						
Female	-1,23 ***	-1,72	-0,75	-0,87 ***	-1,29	-0,45
Male (*ref*)						
**Country**						
The United Kingdom	-2,09 ***	-2,76	-1,42	-0,77 **	-1,35	-0,18
Greece	-0,32	-0,89	0,25	-0,43	-0,93	0,07
Croatia	-1,89 ***	-2,57	-1,20	-1,91 ***	-2,50	-1,32
The Netherlands	-1,54 ***	-2,14	-0,94	0,54 *	0,02	1,07
Spain (*ref*)						
**Level of education**						
Primary or less	-0,99 *	-1,80	-0,19	-0,87 *	-1,57	-0,17
Secondary or equivalent	0,09	-0,69	0,87	-0,58	-1,26	0,10
Tertiary or higher (*ref*)						
**Living situation**						
Living alone	0,26	-0,22	0,75	0,57 **	0,15	0,99
Living with others (*ref*)						
**Alcohol risk**						
No (*ref*)						
Yes	0,87 ***	0,41	1,32	0,49 *	0,09	0,88
**Physical activit**y						
< once a week	-4,02 ***	-4,48	-3,55	-1,75 ***	-2,16	-1,34
≥ once a week (*ref*)						
**Smoking**						
No (*ref*)						
Yes	0,13	-0,32	0,59	-0,10	-0,49	0,30
**Multi-morbidity**						
No (*ref*)						
Yes	-1,74 ***	-2,20	-1,29	-0,13	-0,53	0,26
Adjusted R square, %	31.0			33.9		

PCS-12=12-item Physical Component Summary, with higher scores indicating higher levels of health
MCS-12=12-item Mental Component Summary, with higher scores indicating higher levels of health
Std-β=standardized beta
ref=reference category
* P⩽0.05, ** P⩽0.01, *** P⩽0.001, P-values are based on standardized multivariable linear regression models.

Table S3 *P*-values for interactions between socio-demographic variables and loneliness on PCS-12 and MCS-12

	PCS-12	MCS-12
	***P*-value**	***P*-value**
loneliness*age	0.093	0.545
loneliness*sex	0.069	0.009
loneliness*country	0.156	0.091
loneliness*level of education	0.066	0.087
loneliness*living situation	0.243	0.874
emotional loneliness*age	0.750	0.728
emotional loneliness*sex	0.419	0.222
emotional loneliness*country	0.000*	0.000*
emotional loneliness*level of education	0.078	0.182
emotional loneliness*living situation	0.919	0.090
social loneliness*age	0.994	0.700
social loneliness*sex	0.225	0.020
social loneliness*country	0.020	0.320
social loneliness*level of education	0.710	0.564
social loneliness*living situation	0.352	0.991

PCS-12=12-item Physical Component Summary, with higher scores indicating higher levels of health

MCS-12=12-item Mental Component Summary, with higher scores indicating higher levels of health

UNIANOVA was adopted for interaction analyses with correction of covariates.

After applying Bonferroni correction for multiple testing, the *P*-value equaled P=0.05/30=0.002.

*p<0.002

Table S4. Association between emotional loneliness and Health-Related Quality of Life stratified by country.

Items	PCS-12 The United Kingdom	PCS-12 Greece	PCS-12 Croatia	PCS-12 The Netherlands	PCS-12 Spain	MCS-12 The United Kingdom	MCS-12 Greece	MCS-12 Croatia	MCS-12 The Netherlands	MCS-12 Spain
**Emotional loneliness** (*Yes versus No*)	-3.07*	-2.37	-4.59***	-4.52**	-2.49*	-8.60***	-7.47***	-7.25***	-11.46***	-10.23***
**Age**	-0.06	-0.31*	-0.19	-0.66***	-0.25**	0.15*	-0.03	-0.16	0.23*	0.05
**Sex**										
Female	-1.87*	0.02	-1.51	-4.66**	-3.39**	-1.63*	-3.11**	-1.91	0.24	-1.57
Male (*ref*)										
**Level of education**										
Primary or less	0.26	-2.15	-5.67*	1.66	-2.18	-2.22	-2.57	-5.84*	-0.22	-0.95
Secondary or equivalent	-0.82	1.39	-3.08	4.66	0.38	-2.06	0.24	-2.71	0.12	-1.11
Tertiary or higher (*ref*)										
**Living situation**										
Living alone	-0.60	-1.35	1.48	1.80	0.94	1.71*	3.44*	1.58	0.29	-0.27
Living with others (*ref*)										
**Alcohol risk**										
No (*ref*)										
Yes	1.66	3.97**	1.03	1.93	1.62	-0.16	-0.17	1.72	0.87	3.48**
**Physical activity**										
< once a week	-12.67***	-9.18***	-8.79***	-5.56***	-7.11***	-2.19**	-2.70*	-7.66***	-2.13	-5.34***
≥ once a week (*ref*)										
**Smoking**										
No (*ref*)										
Yes	2.04	2.61	0.13	-1.25	-1.80	0.38	-1.00	0.87	-0.61	-1.32
**Multi-morbidity**										
No (*ref*)										
Yes	-6.37	-6.06**	-7.23***	-4.72**	-5.13**	0.48	-0.61	-2.05	-1.18	0.29
Adjusted R square. %	31.5	33.1	34.5	26.8	18.7	17.4	29.4	35.9	25.8	24.8

MCS-12=12-item Mental Component Summary, with higher scores indicating higher levels of health
ref=reference category
* P≤0.05, ** P≤0.01, *** P≤0.001, P-values are based on multivariable linear regression models.
